# Semaphorin 4A Maintains Trophoblastic Function via Activating the STAT3 Pathway

**DOI:** 10.3390/biom14070826

**Published:** 2024-07-10

**Authors:** Taotao Hou, Pingping Zhang, Haishen Tian, Yan Luo, Juan Li, Kuo Zhang, Yali Li

**Affiliations:** 1 Graduate School of Hebei North University, Zhangjiakou 075000, China; 2 Department of Reproductive Genetics, Hebei General Hospital, Shijiazhuang 050051, China; 3 Department of Gynecology, Tianjin Beichen Hospital, Tianjin 300400, China; 4 Graduate School of University of Science and Technology Beijing, Beijing 100083, China

**Keywords:** missed abortion, trophoblast, semaphorin 4A, proliferation, migration, apoptosis

## Abstract

The migration, proliferation, and apoptosis of trophoblastic cells play a crucial role in ensuring the effective preservation of pregnancy at the maternal–fetal interface. Any deviations in the structure and function of these cells might potentially result in the development of numerous pregnancy-related disorders, including missed abortion (MA). This study involved the examination of *semaphorin 4A* (*SEMA4A*) expression in missed abortion *(n* = 18) and normal early pregnancy (*n* = 18) villus. The findings of this study indicate a statistically significant decrease in the expression of SEMA4A in the villi of individuals diagnosed with missed abortion, as compared to the control group. The results of our vitro study showed that *SEMA4A* promoted the migration and proliferation of trophoblast cells and inhibited their apoptosis. Subsequent studies have shown that *SEMA4A* may be involved in regulating *p-STAT3/STAT3*, *MMP9*, *bcl-2*, and *BAX* levels. In summary, the findings of this study indicate a correlation between the decreased level of *SEMA4A* in chorionic villi and missed abortion. These results offer novel theoretical insights into the proper implantation and development of *SEMA4A* embryos at the maternal–fetal interface.

## 1. Introduction

Missed abortion (MA) is a term used to describe the cessation of embryonic or fetal development, resulting in the retention of the embryo or fetus within the uterine cavity for an extended period. This condition is characterized by the closure of the cervix and the failure of the pregnancy to be naturally expelled within the expected timeframe. Abortion, a prevalent unfavorable result of pregnancy, significantly impacts the physical and mental well-being of women during their childbearing years [1]. The cause of abortion is multifaceted and diverse. Currently, the identified etiological causes mostly encompass chromosomal abnormalities, infectious agents, immunological factors, endocrine disturbances, maternal influences, environmental factors, and deleterious behaviors. There remains a significant number of unidentified factors contributing to the occurrence of miscarriages [2,3,4]. The process of pregnancy is intricate, involving the coordination and cooperation of various bodily activities. One of the crucial requirements for the maintenance of a healthy pregnancy is the preservation of proper invasion and migration of trophoblast cells. During the initial phase of gestation, trophoblast cells exhibit a high degree of invasiveness as they penetrate the decidual matrix and spiral artery within the maternal uterus. These trophoblasts effectively replace endothelial cells, infiltrate the vascular wall, and facilitate the establishment of an adequate blood supply for the development of both the placenta and the fetus. Additionally, they play a crucial role in establishing the connection between the mother and the fetus, as well as transmitting important signals between the two entities. The cell holds significant importance throughout the initial phase of pregnancy [5,6]. The etiology of abortion has been attributed to the aberrant biological activity of trophoblast cells, which are under the regulation of genes in vivo, as substantiated by previous studies [7,8,9]. The attenuation of trophoblast viability has been linked to many pregnancy diseases, including intrauterine fetal growth limitation, preeclampsia, abortion, and stillbirth [10].

Numerous factors have been identified as being associated with the viability of trophoblast cells at the maternal–fetal interface, encompassing growth hormones, semaphorins, inflammatory factors, and other relevant factors. Semaphorins are a substantial group of proteins, initially recognized as molecules that guide axons in the course of neuronal development [11]. Several academics have conducted verification to confirm the expression of semaphorins at the maternal–fetal interface. These semaphorins play a role in regulating endometrial receptivity in the context of pregnancy [12,13]. *SEMA4A*, a member of the semaphorin IV subgroup, is a transmembrane glycoprotein molecule that plays a role in the regulation of a variety of physiological processes, such as angiogenesis, immunological response, carcinogenesis, and the retinal system [14]. *SEMA4A* has been identified as a participant in various cancer processes, including the facilitation of epithelial-to-mesenchymal transition (EMT) in cancer cells and the contribution to drug resistance. Consequently, it is recognized as an oncoprotein [15,16,17]. Furthermore, *SEMA4A* has been extensively investigated in several pathological conditions, including cancer and immunological disorders [18,19,20]. In addition, *MMP9* is involved in the regulation of immune response by *SEMA4A* [21]; *STAT3* signaling pathway is involved in the regulation of cell proliferation and migration by *SEMA4A* [15]. In addition, *SEMA4A* can inhibit cell apoptosis [15], while *BAX* and *bcl-2* are important regulatory factors involved in the apoptosis process [22]. *SEMA4A*, *BAX*, and *bcl-2* are involved in the regulation of cell apoptosis, and whether or not there exists a relationship between them is a topic that can be further studied. Trophoblast cells and tumor cells share similar properties, specifically the capacity for proliferation, migration, and invasion [23]. *SEMA4D*, a member of the semaphorin IV subgroup, has been observed in human trophoblast and decidual tissue. This receptor has been discovered to stimulate the migration and invasion of trophoblastic cells into the maternal decidua and myometrium. Additionally, it plays a role in the remodeling of uterine spiral arteries and contributes to the maintenance of a healthy pregnancy [13]. Furthermore, it has been discovered that *SEMA3A*, a constituent of the semaphorins family, exerts a significant influence on the process of decidualization. Moreover, a reduction in the expression of *SEMA3A* has been linked to cases of unexplained spontaneous abortion [12]. The aforementioned studies bring forth the significance of *SEMA4A*. The functional role of *SEMA4A* in the regulation of trophoblasts and its potential association with abortion remains uncertain. Based on the aforementioned findings, it is postulated that the aberrant expression of *SEMA4A* may impede the proper functioning of trophoblasts, hence contributing to the manifestation of miscarriage.

**Figure 1:**
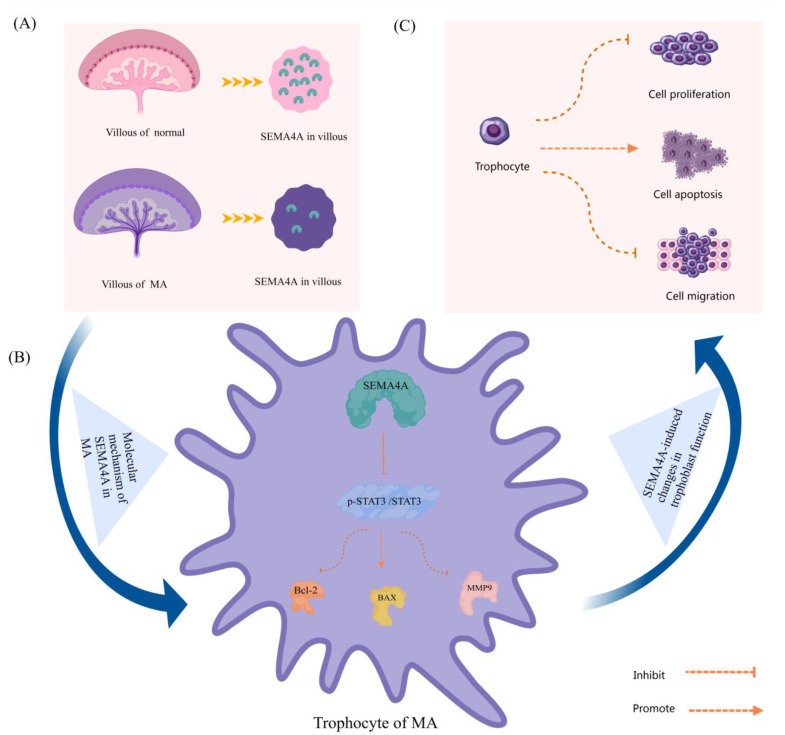
Expression and mechanism of SEMA4A in villus tissue of missed abortion. (**A**) The content of SEMA4A in missed abortion villi was less than that in normal pregnancy villi. (**B**) Molecular mechanism of SEMA4A in missed abortion trophoblastic cells: SEMA4A inhibits bcl-2, MMP9 and promotes BAX by inhibiting p-STAT3 pathway. (**C**) Effect of SEMA4A on trophoblast function in missed abortion: SEMA4A promotes apoptosis and inhibits proliferation and migration of missed abortion trophoblast cells.

This study investigated the expression of *SEMA4A* in the chorionic villi of MA and analyzed its impact on trophoblasts at the interface between the mother and fetus for the first time (Figure 1). This study aimed to assess and compare the expression levels of *SEMA4A* in placental villi between individuals with normal early pregnancy and those diagnosed with MA. Furthermore, we investigated the impact of the substance on trophoblast cells and delved into the underlying process. The data presented in this study offer substantiation for the association between the downregulation of *SEMA4A* in chorionic villi and the occurrence of MA.

## 2. Materials and Methods

### 2.1. Patients and Clinical Samples

The present study received ethical approval from the ethics committee of Hebei General Hospital, and written informed consent was obtained from all participants before the collection of samples. The present study comprised a cohort of 36 primigravid women in the early stages of pregnancy who underwent negative pressure suction for induced abortion at Hebei General Hospital throughout the period spanning from September 2023 to November 2023. Based on the pregnancy outcome, the participants were categorized into two groups: the normal pregnancy group, also referred to as the control group, and the missed abortion group, abbreviated as the MA group, each consisting of 18 cases. The inclusion criteria of the MA group were as follows: (a) estimated gestational age ≤ 12 weeks based on B-ultrasound and last menstrual cycle and (b) B-scan ultrasonography suggesting intrauterine pregnancy, which met the diagnostic criteria for embryo termination (head and hip length ≥7 mm, no fetal heart beat; the mean diameter of the gestational sac was ≥25 mm, and no embryo was found; no yolk sac was seen, and no embryo and fetal heart beat were seen after 2 weeks; the yolk sac was visible, and no fetal heart beat was observed after 11 days). The inclusion criteria of the control group were as follows: (a) no symptoms of threatened abortion; (b) B-scan ultrasound indicates intrauterine pregnancy, pregnancy sac can be seen, consistent with the gestational week, fetal bud and the original cardiac tube can be seen, and no other abnormal conditions exist, such as uterine effusion. The common exclusion criteria encompassed the following: (a) the presence of symptoms related to endocrine or metabolic disorders, such as hyperthyroidism and diabetes; (b) the presence of karyotype abnormalities; (c) the identification of infection through standard leucorrhea examination; and (d) the presence of uterine abnormalities. Following the acquisition of the patient’s informed consent, the collection of chorionic villi samples was conducted, and subsequent thorough washing was performed using sterile saline solution. The villous tissues were promptly collected subsequent to the surgical procedure. One section of the tissues was preserved using a 4% paraformaldehyde solution for subsequent paraffin embedding in blocks, while the other portion was maintained in liquid nitrogen and then put into the refrigerator at −80 °C.

### 2.2. Cell Culture and Treatment

Human first-trimester trophoblast/simian virus (HTR-8/SVneo) (ZQ 0482) cells used in this study were obtained in April 2023 from Shanghai Zhong Qiao Xin Zhou Biotechnology Co., Ltd. of Shanghai, China. These cells were cultured in a 1640 culture medium (Gibco, Thermo Fisher Technologies LTD of Guangzhou, China) supplemented with 10% fetal bovine serum (FBS, Guangdong Oricell Biotechnology Co., Ltd. of Guangdong, China) and 1% penicillin–streptomycin solution (Solarbio, Beijing Solarbio Technology Co., Ltd. of Beijing, China). These cells were cultivated at a temperature of 37 °C in an environment containing 5% CO_2_. The SEMA4A knockdown experiment utilized SEMA4A siRNA and a negative control (si-NC) obtained from Guangzhou RiboBio Co., Ltd. of Guangdong, China. For SEMA4A overexpression, pcDNA3.1-SEMA4A and a negative control (pcDNA3.1-NC) were acquired from Hunan Keai Medical Devices Co., Ltd. of Hunan, China. The HTR-8/SVneo cells were transfected using Lipofectamine 2000 (Invitrogen, Thermo Fisher Technologies LTD of Guangzhou, China) according to the manufacturer’s instructions.

### 2.3. Cell Proliferation Assay

Cell proliferation was evaluated by employing the CCK-8 (APExBIO, APExBIO Technology LLC of Houston, Houston, TX, USA). HTR-8/SVneo cells were seeded onto 96-well plates at a density of 2 × 103 cells per well and subjected to various experimental treatments. At the specified time intervals (0, 24, 48, and 72 h), a 10% concentration of CCK-8 was introduced into every well. Following an extended incubation period of 4 h, the absorbance at a wavelength of 450 nm was quantified using a microplate reader. Three wells were designated as replicates for each group.

### 2.4. Transwell Migration Assay

Cellular migration ability was assessed using a 24-well plate Transwell insert (8-µm pore size) (Corning, Shanghai Muchen Biotechnology Co., Ltd. of Shanghai, China). Briefly, HTR-8/SVneo cells at a density of 2 × 10^5^ were cultured in the upper chamber of each insert using a 200 µL FBS-free 1640 medium. A volume of 650 µL of 1640 medium supplemented with 10% fetal bovine serum (FBS) was introduced into the lower chamber. The Petri dish was positioned within an incubator set at a temperature of 37 °C, with a controlled atmosphere containing 5% carbon dioxide, for a duration of 28 h. Subsequently, the cells that had penetrated the lower chamber were immobilized using a 4% paraformaldehyde solution, subjected to staining with a 0.1% crystal violet solution and subsequently assessed for quantification. The mean cell count obtained from five distinct fields with a magnification of 200× was documented.

### 2.5. Wound Healing Assay

The migration ability of trophoblasts was assessed using the wound healing assay. Trophoblast cells were seeded in a 6-well plate chamber at a density of 5 × 10^5^ cells per well in fresh medium and incubated for 24 h. Once the cells reached 80–90% confluence, a scratch was created across the cell surface using a 200 μL pipette tip. Floating debris was removed using phosphate-buffered saline (PBS), and the wound was immediately photographed (0 h). Subsequently, the cells were cultured in serum-free medium. After 24 h, the wounds were photographed again to quantify the extent of wound healing. The sizes of the gaps were measured using Image J software (v.1.46, National Institute of Health of Bethesda, Bethesda, MD, USA).

### 2.6. Flow Cytometry Analysis

The Annexin V-FITC/PI Apoptosis Detection Kit (Elabscience, Wuhan Elabscience Biotechnology Co., Ltd. of Wuhan, China) was utilized to identify cell apoptosis. In summary, the HTR-8/SVneo cells that underwent transfection were gathered and subsequently rinsed with PBS. Following resuspension in a 1× binding buffer, the cells were subjected to staining with Annexin V-FITC and PI at room temperature, while being kept in a dark environment. Subsequently, the apoptosis rate of HTR8/SVneo cells was assessed through the utilization of a flow cytometer (BD FACSAria III, Jiangsu Kenerfei experimental instrument trading Co., Ltd. of Jiangsu, China). Cells that were positive for Annexin V-FITC and negative for propidium iodide (PI) were identified as early apoptotic cells, whereas those positive for both Annexin V-FITC and PI were classified as late apoptotic cells. The cumulative apoptosis rate was determined by adding the rates of early and late apoptosis.

### 2.7. Western Blot Analysis

The extraction of total protein was performed on either cells or 50 mg of tissues using RIPA lysis solution supplemented with a protease and phosphatase inhibitor cocktail (Servicebio, Wnhan Servicebio Biotechnology Co., Ltd. of Wuhan, China). The cells were suspended in RIPA buffer and incubated on ice for 30 min. The quantification of cell lysis buffer, which contained the total protein, was performed using a BCA Protein assay kit manufactured by Beijing Solarbio Technology Co., Ltd. of Beijing, China. A total of 60 micrograms (μg) of protein was utilized for the purpose of conducting SDS-PAGE electrophoresis. The protein was separated using SDS-PAGE gel and subsequently deposited onto a PVDF membrane. Following a 40 min incubation with a protein-free fast blocking solution, the membrane was subsequently exposed to primary antibodies: SEMA4A (27359-1-aP, 1:1000, Proteintech, Wuhan Proteintech Biotechnology Co., Ltd. of Wuhan, China), β-Actin (GB15003, 1:1500, Servicebio, Wnhan Servicebio Biotechnology Co., Ltd. of Wuhan, China), STAT3 (R22785, 1:750, ZENBIO, China), and p-STAT3 (Ser727) (R25804, 1:750, ZENBIO, Chengdu ZENBIO Biotechnology Co., Ltd. of Chengdu, China), as well as the MMP9 antibody (380831, 1:1000, ZENBIO, Chengdu ZENBIO Biotechnology Co., Ltd. of Chengdu, China), bcl-2 antibody (381702, 1:750, ZENBIO, Chengdu ZENBIO Biotechnology Co., Ltd. of Chengdu, China), and BAX antibody (E-AB-10049, 1:1000, Elabscience, Wuhan Elabscience Biotechnology Co., Ltd. of Wuhan, China). After left to incubate overnight in the primary antibody, the membrane underwent a total of three washes using TBST solution, with each wash lasting for 10 min. Following the washing step, the membrane was further subjected to incubation with a secondary antibody, HRP-conjugated goat anti-rabbit IgG (H + L), at a dilution of 1:8000 (BF03008X, Biodragon, Suzhou Biodragon Immunotechnology Co., Ltd. of Suzhou, China) for 1 h at room temperature. Subsequently, the membrane underwent three washes with TBST, and the protein bands were detected through the utilization of an enhanced chemiluminescence kit (Beijing Solarbio Technology Co., Ltd. of Beijing, China) and captured via photography on the chemiluminescence imaging system. This experiment performed the experiment with 10% SDS-PAGE. The densitometry analysis was conducted using the Image J program (v.1.46, National Institute of Health of Bethesda, Bethesda, MD, USA).

### 2.8. Quantitative Real-Time PCR

The extraction of total RNA from treated cells was performed using TRNzol reagent (TIANGEN, Beijing TIANGEN Biotechnology Co., Ltd. of Beijing, China) in accordance with the instructions provided by the manufacturer. The reverse transcription procedure was performed using the FastKing RT Kit (with gDNase) manufactured by Beijing TIANGEN Biotechnology Co., Ltd. of Beijing, China. The reverse transcription polymerase chain reaction (RT-PCR) was conducted using a SuperReal PreMix Plus (SYBR Green) kit manufactured by Beijing TIANGEN Biotechnology Co., Ltd. of Beijing, China. The amplification and detection of the PCR products were carried out on a 7500 detection system provided by Life Technologies, Singapore. The internal reference β-Actin was used, and the relative gene expression was determined using the 2^−ΔΔCt^ technique. The primers were designed by primer 5, and the primer sequences are provided in Table 1.

### 2.9. Immunohistochemistry

The villous tissues, which had been fixed in paraffin, were sliced into sections that were 4 μm thick. These sections were then subjected to dehydration using a series of ethanol solutions with increasing concentrations. The endogenous peroxidase activity was inhibited by treating the sample with a 3% hydrogen peroxide solution for 25 min. Additionally, any nonspecific binding was prevented by blocking the sample with a 5% bovine serum albumin (BSA) solution for 30 min. Subsequently, the samples were subjected to incubation with a primary rabbit anti-human *SEMA4A* antibody (1:50, Proteintech, Wuhan Proteintech Biotechnology Co., Ltd. of Wuhan, China) for an overnight period at a temperature of 4 °C. The slices underwent three washes with PBS and were subsequently treated with secondary antibodies for 50 min. The detection of the reaction was accomplished using 3,3′-diaminobenzidine (DAB), and subsequently, the sections were counterstained with hematoxylin. Three visual fields were chosen, and the staining was examined using a microscope at a magnification of 200×. The photos were analyzed using the software Image J (v.1.46, National Institute of Health of Bethesda, Bethesda, MD, USA).

### 2.10. Hematoxylin–Eosin Staining

The villus tissues were subjected to overnight fixation using a 4% paraformaldehyde solution. Following this, the tissues underwent paraffin embedding treatment, and 4-micron slices were produced for subsequent experimental procedures. The tissue sections underwent deparaffinization using xylene, followed by hydration using alcohol. The sections were subjected to immersion in hematoxylin (Solarbio, Beijing Solarbio Technology Co., Ltd. of Beijing, China) for 5 min at ambient temperature. Following this, they were rinsed with tap water, immersed in a differentiation solution for a brief period, rinsed once more with tap water, subjected to a reversal of the blue color using an anti-blue solution, and then rinsed with running water. Following a 5 min staining period with eosin, the sections underwent a 5 min dehydration process using anhydrous ethanol (I, II, III), followed by a 5 min permeabilization step using xylene (I, II). Subsequently, the slices were treated with a neutral resin and were subsequently examined using an optical microscope (Precise, Beijing Precise Instrument Co., Ltd. of Beijing, China).

### 2.11. Statistical Analysis

Statistical analyses were performed by SPSS 25.0, and statistical figures were created by GraphPad Prism 9.5. The measurement data were tested by Shapiro–Wilk for the normality test. If the data obeyed normal distribution, they were expressed as mean ± standard deviation (SD). *t*-test was used for comparison between the two groups, analysis of variance was used for repeated measurement data, and the Bonferroni correction method was used for multiple comparisons afterwards. If the data do not obey normal distribution, they were expressed by M (P25, P75). *p* < 0.05 was considered to be statistically significant.

## 3. Results

### 3.1. Comparison of Clinical Data between the Two Groups

Compared with the control group, there were no significant differences in the age, gestational age, BMI, pregnancy, and parturitive times of the MA group (*p* > 0.05), indicating that the basic conditions of the two groups were the same and that the two groups were comparable, as shown in Table 2.

### 3.2. SEMA4A Is Expressed at Low Levels in the Villous Tissues of MA Patients

**Figure 2:**
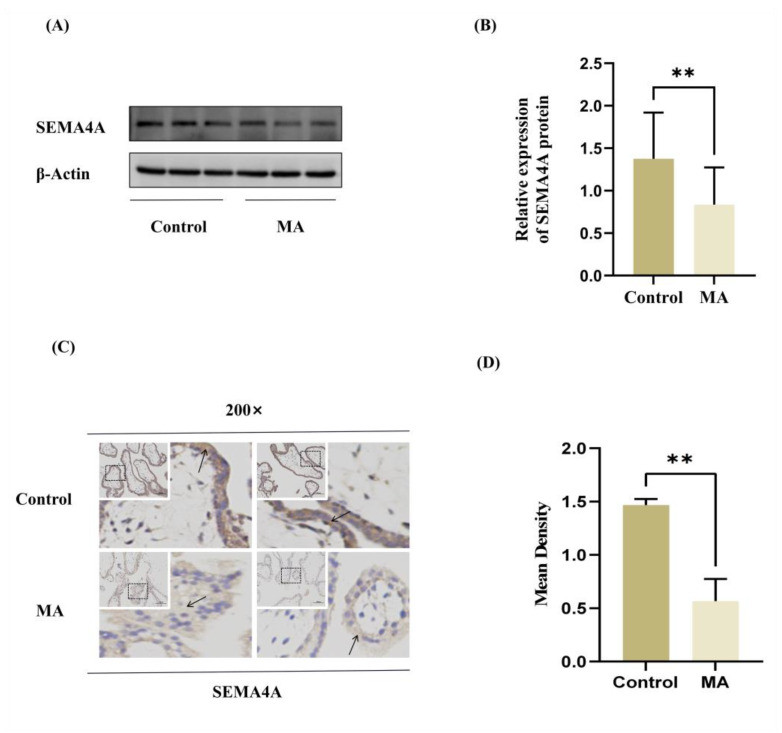
The expression of *SEMA4A* in human villous tissues from the MA and the control groups. (**A**,**B**) Relative expression levels of *SEMA4A* protein normalized to *β-Actin* in the MA (*n* = 18) and control groups (*n* = 18) by Western blotting. (**C**,**D**) Immunohistochemical staining of *SEMA4A* in villous tissues from the MA (*n* = 5) and control (*n* = 5) groups. Black arrow: *SEMA4A* distribution region. The negative control was the control group. Data represent mean ± SD. Scale bars: 100 μm. ** *p* < 0.01. Original Western blot images are available in Supplementary Materials.

To study *SEMA4A* expression in human villous tissues, villous tissues were obtained from women with missed abortion (MA group) and women with normal early pregnancy (control group). Table 2 displays the clinical information. Subsequently, the quantification of *SEMA4A* expression in villous tissues from both groups was performed utilizing Western blot. The findings of this study indicate that there was a decrease in *SEMA4A* protein expression in the group with MA (Figure 2A,B). In addition, an immunohistochemistry study was conducted to ascertain the distribution of *SEMA4A* in villous tissues. Notably, a diminished *SEMA4A* staining pattern was found in the MA group (Figure 2C,D). The findings presented in this study indicate that there may be a certain correlation between the reduced expression of *SEMA4A* and MA.

### 3.3. Histomorphological Observation of Villi in Patients with Different Abortions

**Figure 3:**
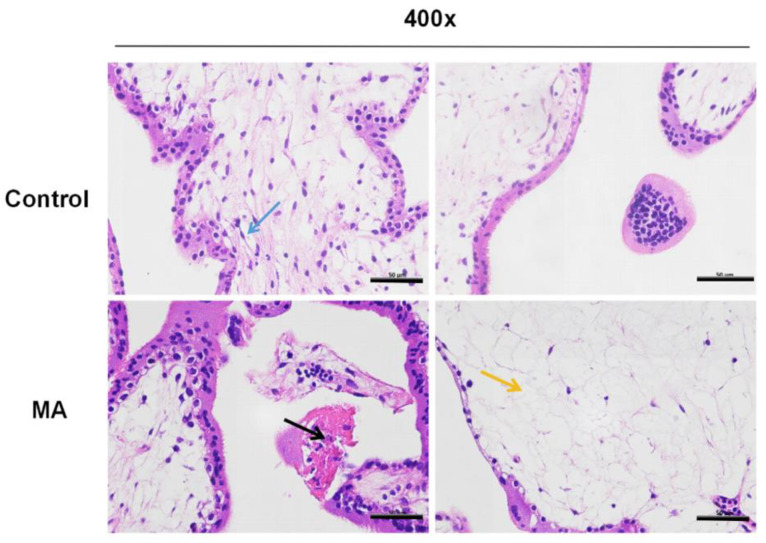
Histopathological changes in villous tissue were detected by HE staining. The control group: the morphology and structure of trophoblast cells were normal, with clear boundaries and abundant cytoplasm; no edema was observed in the villous interstitium; and the vascular structure was clear (blue arrow). The MA group: A small amount of villous epithelial cell necrosis, nuclear fragmentation or dissolution, cytoplasmic eosinophilic enhancement (black arrow), intrastromal edema, and loose structural arrangement (yellow arrow) were observed in the villi. Scale bars: 50 µm.

The chorionic villi from both groups were subjected to hematoxylin and eosin (HE) staining, and further examination of the pathological morphology was conducted using a light microscope. The researchers noticed the following observations: The normal early pregnancy group exhibited normal morphology and structure of trophoblast cells, with clear boundaries and abundant cytoplasm. No edema was observed in the villous interstitium and the vascular structure was clear (blue arrow). No obvious inflammatory cell infiltration was observed. However, within the MA group, a small amount of villous epithelial cell necrosis, nuclear fragmentation or dissolution, and cytoplasmic eosinophilic enhancement (black arrow) were observed in placental tissue. Locally, there was mild villi edema and loose interstitial arrangement (yellow arrow). No obvious inflammation was observed. The pathomorphology of villi in the MA group exhibited abnormalities when compared to the control group (Figure 3).

### 3.4. SEMA4A Suppresses HTR-8/SVneo Cell Apoptosis

**Figure 4:**
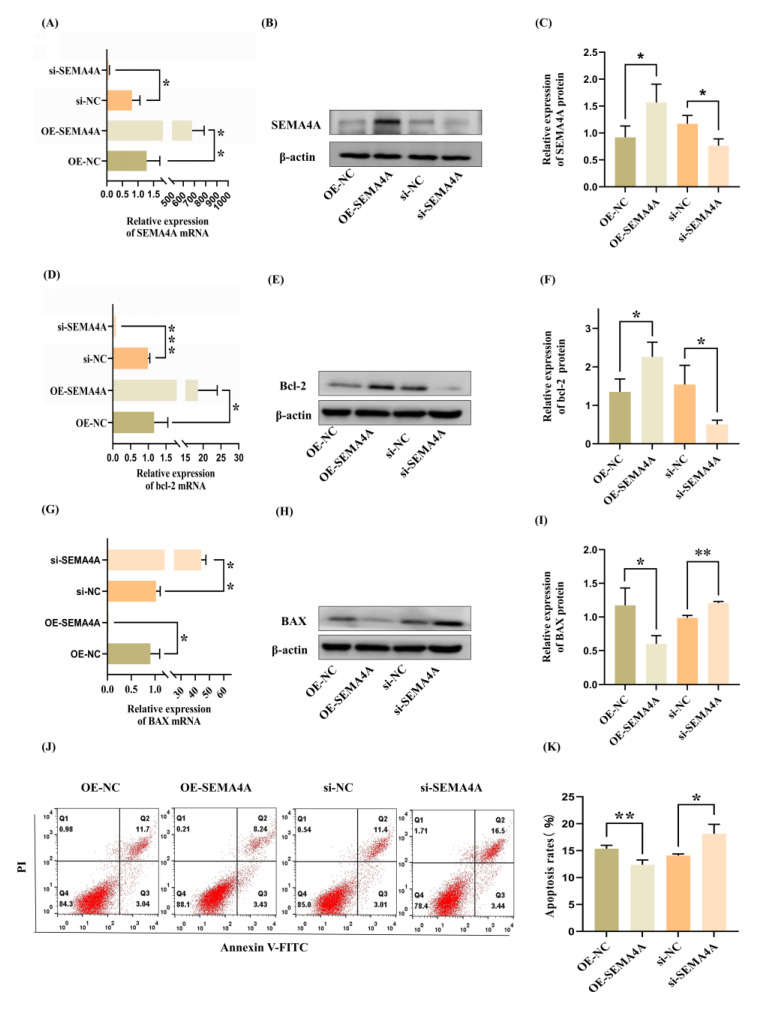
*SEMA4A* decreased HTR-8/SVneo cell apoptosis. (**A**–**C**) *SEMA4A* overexpression and knockdown were confirmed by qRT-PCR (*n* = 3) and Western blot (*n* = 3) analysis. (**D**–**I**) Representative expression of qRT-PCR (*n* = 3) and Western blot (*n* = 3) analysis for *bcl-2* and *BAX* in HTR-8/SVneo cells. (**J**,**K**) The ratio of apoptotic cells (*n* = 3). (**A**,**D**,**G**) Different groups of HTR-8/SVneo are presented on the y-axis for the qRT-PCR; the mean ± SD of 2^−ΔΔCt^ for each group is presented on the x-axis for the qRT-PCR. Data represent mean ± SD. * *p* < 0.05; ** *p* < 0.01; *** *p* < 0.001. The experiment was repeated three times. Original Western blot images are available in Supplementary Materials.

To examine the impact of *SEMA4A* on the biological characteristics of trophoblast cells, *SEMA4A* overexpression was induced in HTR-8/SVneo cells through transient transfection using *SEMA4A* expression plasmids. Additionally, particular siRNA molecules were employed to decrease the level of *SEMA4A* in HTR-8/SVneo cells. The verification of the efficiency of *SEMA4A* overexpression and knockdown was conducted by the utilization of qRT-PCR and Western blot analysis (Figure 4A–C). Subsequently, the utilization of flow cytometry analysis was conducted to assess the impact of *SEMA4A* on trophoblast cell death. The knockdown of *SEMA4A* resulted in a notable rise in the apoptotic rate of HTR-8/SVneo cells, whereas the overexpression of *SEMA4A* had a strong inhibitory effect on cell apoptosis (Figure 4J,K). *BAX* and *bcl-2* are representative members of the B-cell lymphoma 2 (*bcl-2*) protein family, exhibiting anti-apoptotic and pro-apoptotic properties, respectively. These proteins play a crucial role in maintaining a finely tuned equilibrium within normal cellular processes. The level of *bcl-2* was significantly increased in cells overexpressing *SEMA4A*, while it was decreased in *SEMA4A*-silenced HTR-8/SVneo cells (Figure 4D–F). In contrast, the level of *BAX* was significantly decreased in cells overexpressing *SEMA4A*, while it was increased in *SEMA4A*-silenced HTR-8/SVneo cells (Figure 4G–I). The findings demonstrated the impact of *SEMA4A* on the inhibition of cellular death.

### 3.5. SEMA4A Induces Cell Proliferation and Promotes the Migration of HTR-8/SVneo Cells

**Figure 5:**
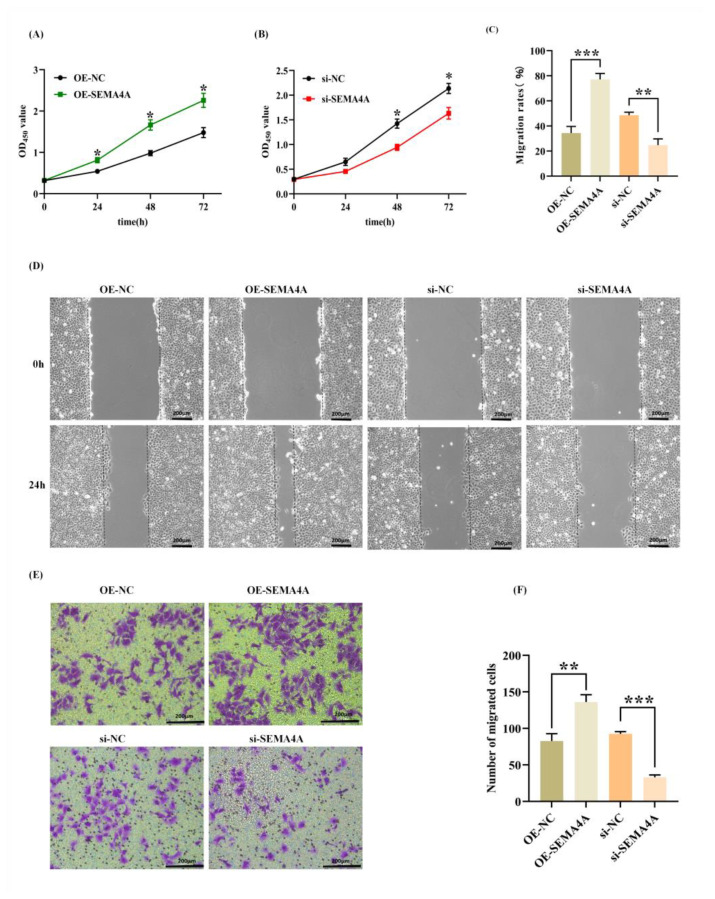
*SEMA4A* increased HTR-8/SVneo cell proliferation and migration. (**A**,**B**) CCK-8 assay (*n* = 3) was conducted to analyze the proliferation of *SEMA4A*-overexpressed/silenced HTR-8/SVneo cells. (**C**,**D**) Wound healing scratches (*n* = 3) were imaged immediately and 24 h after initial scratch time to quantify relative migration. (**E**,**F**) The Transwell migration (*n* = 3) assay was used to test the ability of cells to migrate. Scale bar = 200 μm. Data represent mean ± SD. * *p* < 0.05; ** *p* < 0.01; *** *p* < 0.001.

The CCK-8 assay was conducted to assess the proliferation of trophoblast cells. It was noted that the overexpression of *SEMA4A* resulted in the proliferation of HTR-8/SVneo cells. Conversely, a notable reduction in cell proliferation was detected in *SEMA4A*-depleted HTR-8/SVneo cells (Figure 5A,B). These findings suggest that *SEMA4A* has a stimulating effect on trophoblast cell proliferation. Subsequently, an estimation is made regarding the migratory capacity of HTR-8/SVneo. Following the scratch test, it was shown that the overexpression of *SEMA4A* resulted in a more rapid closure of wounds in comparison to the control group. On the other hand, the suppression of *SEMA4A* expression resulted in the inhibition of trophoblast cell migration (Figure 5C,D). The results of the Transwell migration experiment indicated a significant increase in the number of migrated cells belonging to the *SEMA4A* overexpression group in comparison to the control group. Conversely, the *SEMA4A* knockdown group exhibited a lower number of migrated cells compared to the control group (Figure 5E,F). These findings were consistent with the results obtained from the wound healing experiment. The aforementioned findings indicate that SEMA4A facilitates the proliferation and migration of HTR-8/SVneo cells.

### 3.6. SEMA4A Affects MA Pathogenesis by Activating the STAT3 Signaling Pathway

**Figure 6:**
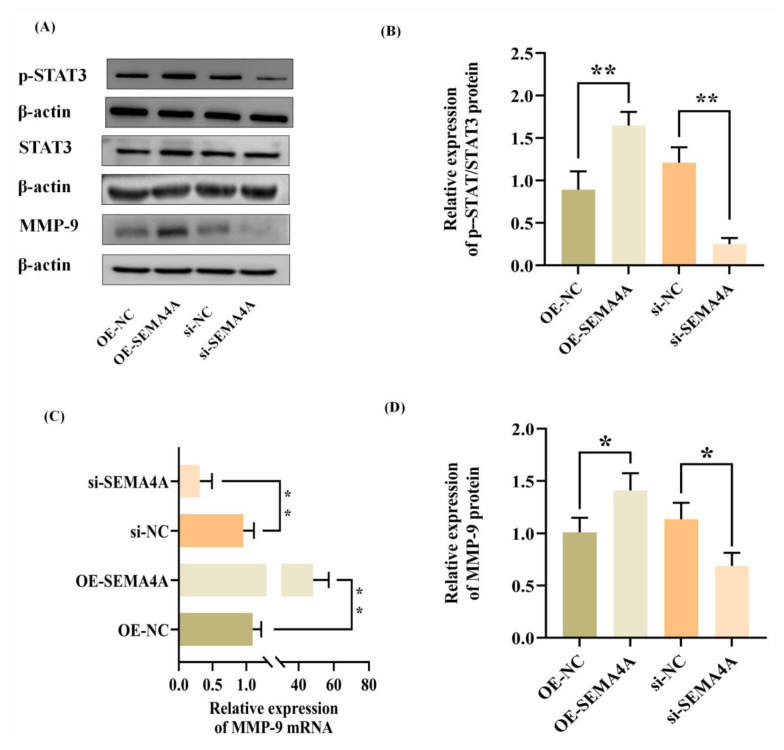
Effect of *SEMA4A* on *p-STAT3/STAT3* and *MMP9* in trophoblast. (**A**,**B**) Representative images and bar graph of Western blot (*n* = 3) analysis for *p-STAT3/STAT3* in HTR-8/SVneo cells. (**A**,**C**,**D**) *MMP-9* mRNA and protein expression in HTR-8/SVneo cells transfected with *SEMA4A* siRNAs or *SEMA4A* overexpression vectors by qRT-PCR (*n* = 3) and Western blot (*n* = 3) analysis. (C) Different groups of HTR-8/SVneo are presented on the y-axis for the qRT-PCR; the mean ± SD of 2^−ΔΔCt^ for each group is presented on the x-axis for the qRT-PCR. * *p* < 0.05; ** *p* < 0.01. Original Western blot images are available in Supplementary Materials.

Prior research has indicated a correlation between *SEMA4A* and the *STAT3* pathway [15]. To gain a deeper understanding of the underlying processes by which *SEMA4A* influences trophoblast activity, the experiment investigated the impact of SEMA4A on the *p-STAT3/STAT3*. The level of *SEMA4A* and *STAT3* signaling proteins was observed in trophoblast cells. The findings indicated that the amount of the phosphorylated form of *STAT3* (*p-STAT3*) protein, which plays a crucial role in trophoblast migration in HTR-8/SVneo, remained affected by *SEMA4A*. *p-STAT3/STAT3* was upregulated following transfection with pcDNA-*SEMA4A* and downregulated following transfection with si-*SEMA4A* (Figure 6A,B). Furthermore, it was shown that increasing the level of *SEMA4A* resulted in an upregulation of both mRNA and protein levels of *MMP9*, which are essential for cells to migrate in HTR-8/SVneo. Conversely, the deletion of *SEMA4A* led to a decrease in both mRNA and protein expression levels of MMP9 (Figure 6A,C,D). The results of this study indicate that *SEMA4A* may enhance the migration ability of HTR-8/SVneo through *p-STAT3/STAT3* and *MMP9*.

## 4. Discussion

The proliferation, migration, and invasion of trophoblast cells play crucial roles in the development of the fetus and placenta. It has been widely recognized that the reduction in trophoblast cell proliferation and metastasis is associated with the development of missed abortion [24]. Previous studies have demonstrated that *SEMA4A* plays a role in facilitating the proliferation and metastasis of breast carcinoma [15]. The present investigation has identified *SEMA4A* as a contributing factor to the proliferation and migration of HTR-8/SVneo cells. These findings imply that targeting *SEMA4A* could be a feasible technique for preventing missed abortion.

The findings of this study provide confirmation of the downregulation of *SEMA4A* in placental villi in patients diagnosed with missed abortion. The acquisition of trophoblasts during the early stages of pregnancy poses significant challenges, hence presenting a notable drawback in investigating the intricate mechanisms behind trophoblast invasion. Several researchers have observed that the invasiveness of the HTR-8/SVneo cell line is nearly equivalent to that of primary trophoblast cells when compared to JEG3 and Bewo cell lines. Considering this point, it can be argued that the HTR-8/SVneo cell line possesses characteristics that make it particularly well suited for investigating the physiological and associated molecular mechanisms of trophoblast invasion [25]. Up to now, the HTR-8/SVneo cell line has been extensively employed in research to investigate the biological properties and functions of trophoblast cells [26]. The development of the deliberate termination of pregnancy was found to be associated with preventing cell proliferation and migration in HTR-8/SVneo [27]. In this study, the downregulation of *SEMA4A* was observed to have a significant impact on the viability and migration of HTR-8/SVneo cells. These findings suggest that the decreased level of *SEMA4A* in the placental villi of patients with missed abortion may impede the migration of trophoblast cells into the maternal vessels and decidua. Consequently, this could result in inadequate nutrient supply and subsequent embryo demise, thereby contributing to the occurrence of missed abortion.

Missed abortion is classified as an early abnormal pregnancy characterized by the presence of a deceased embryo or delayed embryoplastic development [28]. The process of cell death in trophoblasts was observed to be enhanced throughout the progression of missed abortion [28]. *Bcl-2* and *BAX* are two genes that are often seen and which are associated with apoptosis. The *bcl-2* protein exerts regulatory control over apoptotic pathways that are dependent on mitochondria, primarily by modulating the permeability of the mitochondrial membrane and regulating the release of components involved in apoptosis. Furthermore, it has been observed that *bcl-2* has the ability to impede cellular apoptosis induced by various causes, leading to a substantial extension of the cell’s development phase and an augmentation of its resistance to apoptotic stimuli [29,30]. The excessive production of the *BAX* gene can impede the activity of the *bcl-2* gene and facilitate the process of apoptosis. Upon receiving the death signal, the *BAX* gene initiates the translocation of the *BAX* protein to the mitochondrial membrane, resulting in the formation of a molecular pore that exhibits cytotoxic effects. This process subsequently leads to mitochondrial malfunction and alterations in permeability, ultimately culminating in apoptosis [31]. Zhao et al. [32] observed that the dysregulation of *BAX* and *bcl-2* expression had a strong association with trophoblast cell death and played a significant role in the development of abortion. The experimental findings indicate that the manipulation of *SEMA4A* expression resulted in alterations in the expression levels of *BAX* and *bcl-2*. In cells where *SEMA4A* expression was suppressed, there was an observed rise in apoptotic levels. Additionally, the levels of *BAX* were found to be significantly elevated, while the levels of *bcl-2* were notably reduced. In contrast, it was shown that in cells overexpressing *SEMA4A*, there was a decrease in apoptotic levels, a large decrease in *BAX* levels, and a marked increase in *bcl-2* levels. There is a suggestion that *SEMA4A* has the potential to impede the intrinsic pathway of apoptosis via the *bcl-2/BAX* molecular switch, which plays a critical role in regulating apoptosis.

Matrix metalloproteinases (MMPs) are a group of proteolytic enzymes that rely on zinc for their activity. In the context of cytotrophoblasts, these cells secrete *MMP9* to facilitate the degradation of the extracellular matrix. This process is crucial for the successful seeding and implantation of the embryo [33]. *MMP9*, also known as matrix metalloproteinase-9, is a collagenase belonging to the type IV collagenase family. It possesses the highest molecular weight and exhibits the most potent effects. *MMP-9* has been found to play a crucial role in various biological processes, including the promotion of capillary angiogenesis, involvement in cellular inflammatory response as an inflammatory mediator, inhibition of cell apoptosis, and facilitation of cell proliferation, migration, and invasion [34]. *MMP9* is closely related to epithelial–mesenchymal transformation (EMT). The etiology of missed abortion is attributed to defects in the epithelial–mesenchymal transition of trophoblast, as indicated by previous research [33]. The findings of this study demonstrated that the overexpression of *SEMA4A* led to a significant upregulation of both mRNA and protein levels of *MMP9* in HTR-8/SVneo cells. Therefore, *SEMA4A* may be involved in the biological behavior changes in trophoblast cells through *MMP9*.

The involvement of *SEMA4A* in pathological processes has been observed through many pathways, such as the *STAT3* pathway [15,35]. The regulation of target genes associated with trophoblast cell invasion has been documented to be influenced by *STAT3* signaling. Furthermore, the invasiveness of trophoblast cells was found to be increased through the phosphorylation of *STAT3* [36]. The reduction in phosphorylated *STAT3* in the decidualization of mouse decidua was observed, which resulted in the impairment of embryo implantation and had a role in the occurrence of miscarriage [37]. Hence, it can be inferred that *p-STAT3* plays a crucial role in the process of embryo implantation and the creation of the placenta [38]. Consequently, it is regarded as a prospective target for addressing the issue of spontaneous abortion [37]. Additionally, the upregulation of *MMP9* mRNA and protein levels in HTR-8/SVneo cells may potentially be linked to the activation of *STAT3*, as shown by a previous study [39]. The findings of our investigation revealed that the overexpression of *SEMA4A* resulted in an augmentation of *p-STAT3* protein level in HTR-8/SVneo cells. Conversely, the inhibition of *SEMA4A* led to a reduction in the observed rise in *p-STAT3* level. We speculate that *SEMA4A* may promote the proliferation and migration of trophoblast cells through the *p-STAT3/STAT3* pathway, but the specific mechanism of action still needs to be explored.

In conclusion, this study showed that *SEMA4A* level was significantly reduced in chorionic villus tissues of individuals diagnosed with MA. Inhibition of *SEMA4A* can induce apoptosis of trophoblast cells and inhibit cell migration and proliferation, while upregulation of *SEMA4A* can inhibit apoptosis of trophoblast cells and promote cell migration and proliferation. The upregulation of *SEMA4A* had a suppressive effect on programmed cell death through the regulation of *BAX* and *bcl-2* gene expression. Additionally, *SEMA4A* may promote trophoblast migration through *p-STAT3/STAT3* expression.

## 5. Conclusions

In summary, *SEMA4A* expression was downregulated in the villi tissue of MA in early pregnancy. *SEMA4A* promoted the proliferation and migration of trophoblast cells and inhibited their apoptosis. *SEMA4A* may be involved in regulating *p-STAT3/STAT3*, *MMP9*, *bcl-2*, and *BAX*. This work has the potential to identify a previously unexplored therapeutic target for the treatment of missed abortion. However, the physiological activities in humans are complex. Further research is needed to verify the role of *SEMA4A* in MA and molecular mechanisms in animal models. Meanwhile, other classical cytokines remain to be investigated.

## Figures and Tables

**Table 1:** Primer sequences.

Genes	Primers	Sequences (5′–3′)	A Product Length	Efficiencies
β-Actin	Forward	CATGTACGTTGCTATCCAGGC	137 bp	100%
	Reverse	CTCCTTAATGTCACGCACGAT		
SEMA4A	Forward	TGGATGGGATGCTCTATTCTGG	132 bp	100%
	Reverse	GCGGAGGAAGTTGTCGGTC		
MMP9	Forward	TGTACCGCTATGGTTACACTCG	155 bp	97%
	Reverse	GGCAGGGACAGTTGCTTCT		
BAX	Forward	CCCGAGAGGTCTTTTTCCGAG	148 bp	99%
	Reverse	CCAGCCCATGATGGTTCTGAT		
Bcl-2	Forward	GGTGGGGTCATGTGTGTGG	146 bp	99%
	Reverse	CGGTTCAGGTACTCAGTCATCC		

**Table 2:** Clinical characteristics of the population.

Groups	Age (Year)	Gestation Age (Day)	BMI (kg/m^2^)	Gestational Times	Birth Times
Normal group *(n* = 18)	23.11 ± 1.53	49.61 ± 5.66	21.66 ± 3.29	1 (1,2)	0 (0,1)
MA group (*n* = 18)	23.50 ± 1.62	50.44 ± 5.18	22.93 ± 1.53	1.5 (1,2)	0 (0,0.25)
t/Z value	−0.741	−0.461	−1.48	−0.858	−0.766
*p* value	0.464 *	0.648 *	0.148 *	0.462 ^#^	0.563 ^#^

*: Independent sample *t*-test; ^#^: Mann–Whitney U test. Continuous variables are presented as mean ± SD, and enumeration data are presented as median (1st quantile, 3rd quantile). BMI: body mass index. *p* value less than 0.05 is considered as statistically significant.

## Data Availability

The datasets generated and/or analyzed during the current study are available from the corresponding author on reasonable request.

## Supplementary Materials

The following supporting information can be downloaded at: https://www.mdpi.com/article/10.3390/biom14070826/s1, Supplementary File: Original Western Blot images.
