# Supplementary material for: Semaphorin 4A Maintains Trophoblastic Function via Activating the STAT3 Pathway
Source: Biomolecules. 2024 Jul 10;14(7):826. doi: 10.3390/biom14070826 (PMC11274653; doi:10.3390/biom14070826)
Supplement: Supplementary file 1 [file biomolecules-14-00826-s001.zip › biomolecules-3056725-supplementary.pdf]

Villous tissue:

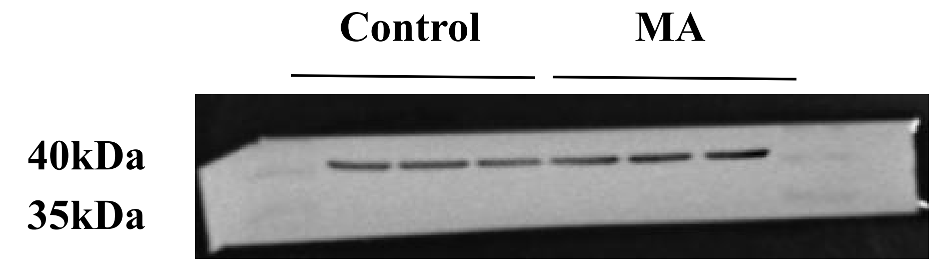

β-Actin：42kDa

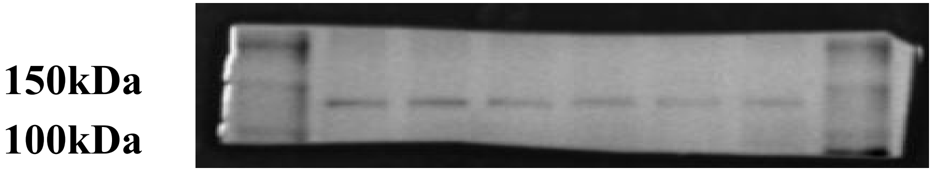

SEMA4A：140kDa

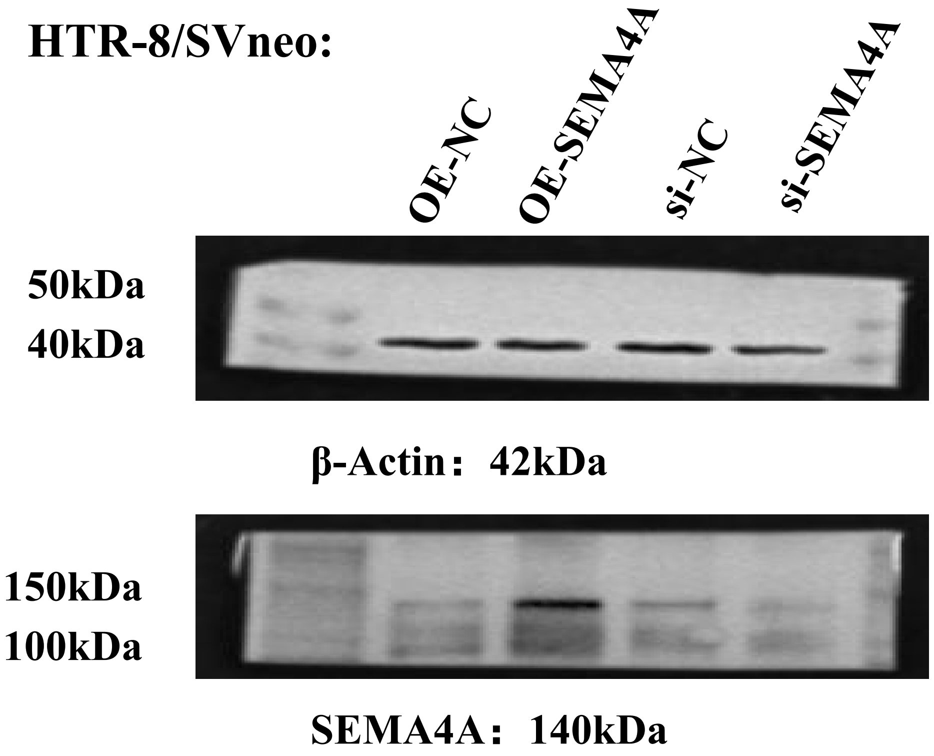
HTR-8/SVneo:
OE-NC
OE-SEMA4A
si-NC
si-SEMA4A
50kDa
40kDa
β-Actin：42kDa
150kDa
100kDa
SEMA4A：140kDa

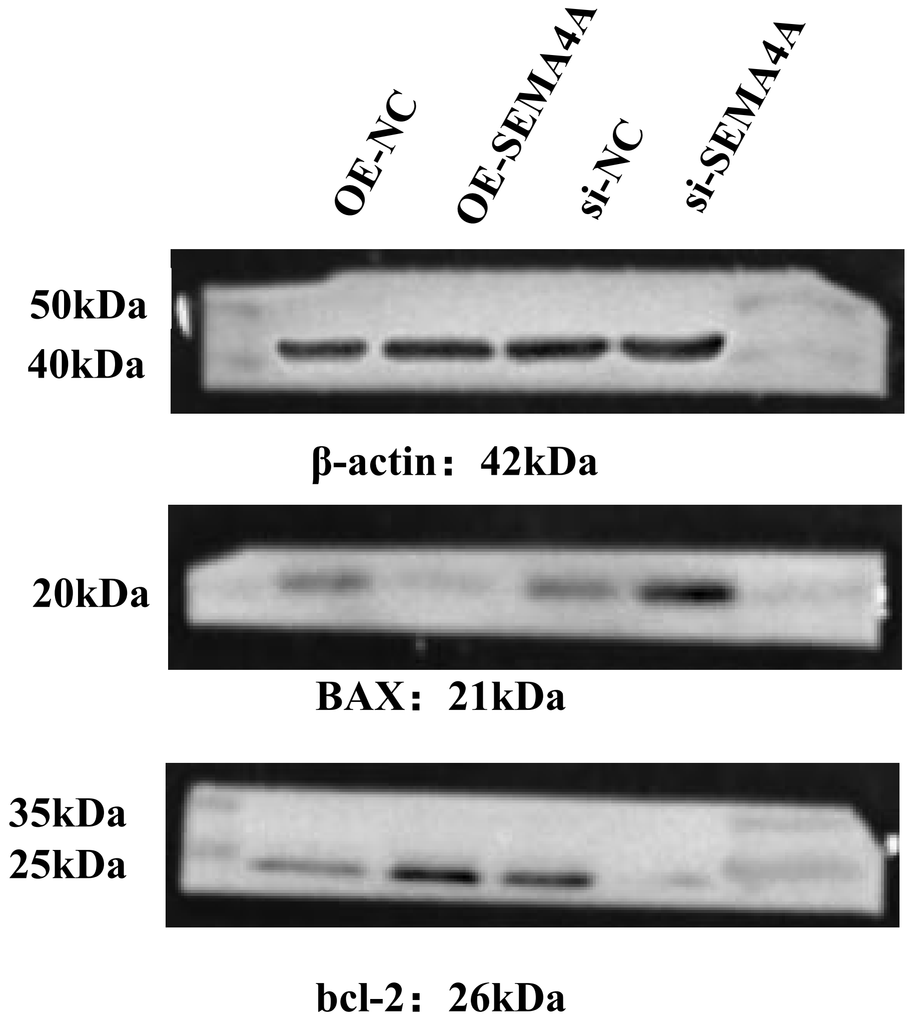
OE-NC
OE-SEMA4A
si-NC
si-SEMA4A
50kDa
40kDa
β-actin：42kDa
20kDa
BAX：21kDa
35kDa
25kDa
bcl-2：26kDa

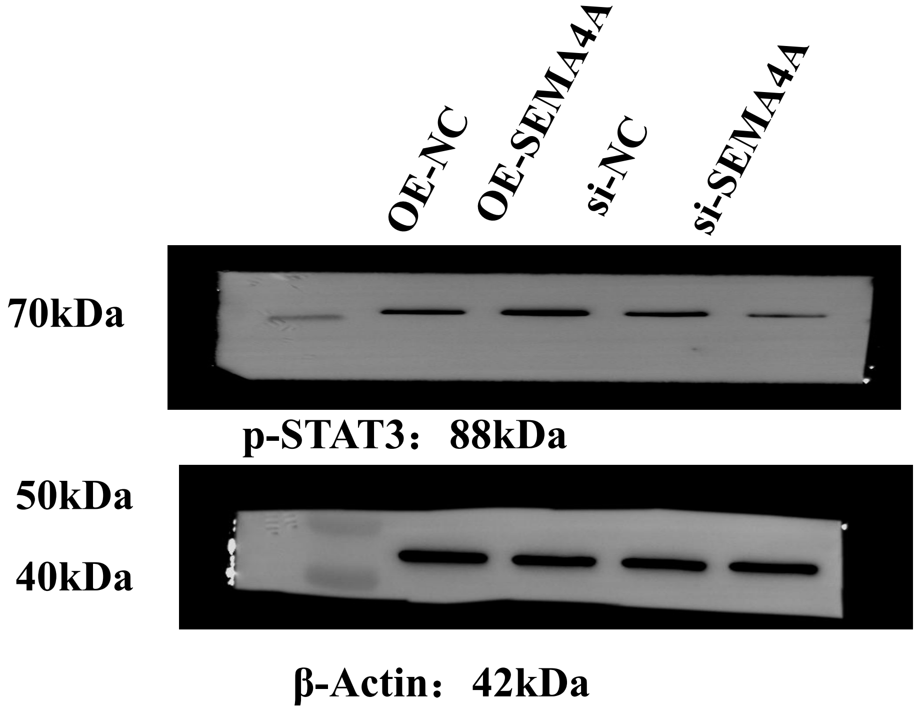
OE-NC
OE-SEMA4A
si-NC
si-SEMA4A
70kDa
p-STAT3：88kDa
50kDa
40kDa
β-Actin：42kDa

HTR-8/SVneo：

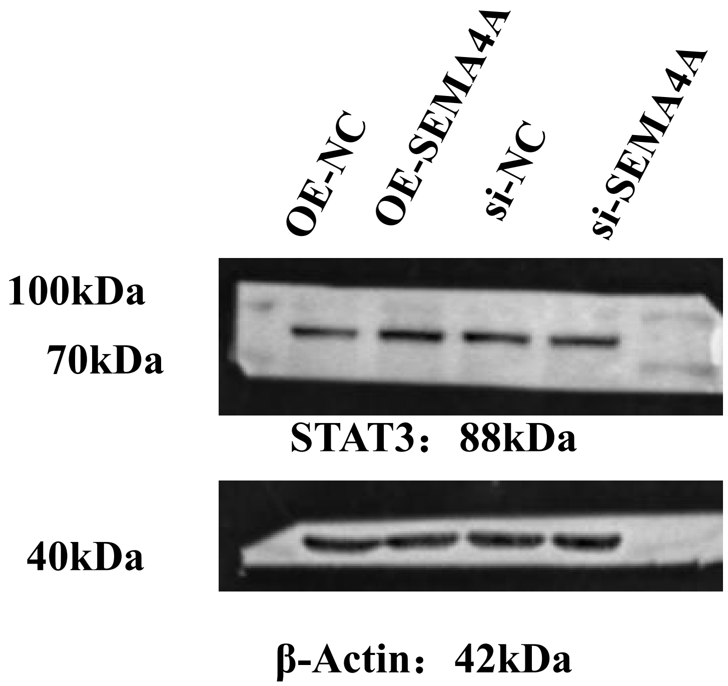
OE-NC
OE-SEMA4A
si-NC
si-SEMA4A
100kDa
70kDa
STAT3：88kDa
40kDa
β-Actin：42kDa

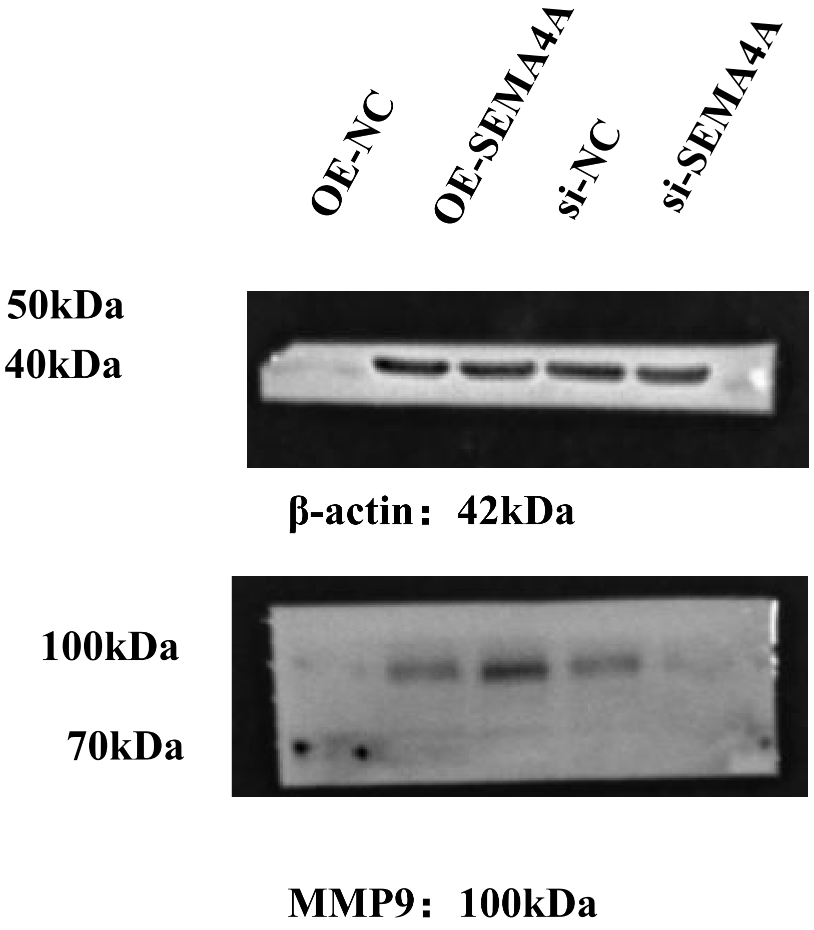
OE-NC
OE-SEMA4A
si-NC
si-SEMA4A
50kDa
40kDa
β-actin：42kDa
100kDa
70kDa
MMP9：100kDa
